# Current Trends in Cytoreductive Surgery (CRS) and Hyperthermic Intraperitoneal Chemotherapy (HIPEC) for Peritoneal Disease from Appendiceal and Colorectal Malignancies

**DOI:** 10.3390/jcm11102840

**Published:** 2022-05-18

**Authors:** Megan M. Harper, Joseph Kim, Prakash K. Pandalai

**Affiliations:** Division of Surgical Oncology, Department of Surgery, University of Kentucky, 800 Rose St., Lexington, KY 40536, USA; megan.harper@uky.edu (M.M.H.); joseph.kim@uky.edu (J.K.)

**Keywords:** cytoreductive surgery (CRS), hyperthermic intraperitoneal chemotherapy (HIPEC), peritoneal surface malignancies (PSM), peritoneal carcinomatosis, colorectal cancer, appendiceal cancer

## Abstract

Peritoneal carcinomatosis (PC) is a poor prognostic factor for all malignancies. This extent of metastatic disease progression remains difficult to treat with systemic therapies due to poor peritoneal vascularization resulting in limited drug delivery and penetration into tissues. Cytoreductive surgery (CRS) and hyperthermic intraperitoneal chemotherapy (HIPEC) are surgical interventions that directly target peritoneal tumors and have improved outcomes for PC resulting from appendiceal and colorectal cancer (CRC). Despite these radical therapies, long-term survival remains infrequent, and recurrence is common. The reasons for these outcomes are multifactorial and signal the need for the continued development of novel therapeutics, techniques, and approaches to improve outcomes for these patients. Here, we review landmark historical studies that serve as the foundation for current recommendations, recent discoveries, clinical trials, active research, and areas of future interest in CRS/HIPEC to treat PC originating from appendiceal and colorectal malignancies.

## 1. Introduction

Although primary peritoneal malignancies are rare, the peritoneum is a common site of metastases or peritoneal carcinomatosis (PC) for organs within the peritoneal cavity. Most commonly, PC arises from appendiceal, colorectal, ovarian, and gastric malignancies [1,2,3]. While less frequent, PC can also arise from gastrointestinal (GI) malignancies from other abdominal organs, including the small bowel, pancreas, gallbladder, and rarely from non-GI and extra-abdominal organs, such as breast, lung, and melanoma [1,2,3,4]. PC is so common with many GI malignancies that it is present at the time of diagnosis in approximately 5–10% of colorectal and 40–70% of appendiceal cancers [5,6]. However, due to non-specific symptoms, delayed presentation, and unreliable or difficult to interpret imaging, the true incidence of PC is likely to be considerably underestimated [2,7]. For instance, in one autopsy study, up to 80% of patients who died from colorectal cancer (CRC) had PC [8]. The high incidence and prevalence of PC makes the discovery of effective therapies of great importance. However, a curative treatment for this disease state remains elusive more than a century after its initial characterization.

Historically, PC was viewed as an inevitable progression of GI malignancies, with an abysmal 5-year survival rate of <5%. Once deemed untreatable, advancements throughout the last century in systemic chemotherapy, cytoreductive surgery (CRS), and hyperthermic intraperitoneal chemotherapy (HIPEC) have improved the survival and quality of life for patients with PC [9]. The surgical technique of CRS involves removing all macroscopic disease. HIPEC, the administration of chemotherapy to the peritoneal cavity to treat microscopic disease, is often combined with CRS. Heat is an integral component of HIPEC to promote drug penetration and facilitate synergistic cytotoxicity with chemotherapies. Although CRS and HIPEC are now performed routinely around the world, indications, techniques, and protocols vary greatly [10,11]. Here, we will review the advances in CRS/HIPEC methodology, techniques, and research to treat PC arising from the most common GI malignancies, appendiceal and colorectal, while discussing our own institutional experience conducting HIPEC-focused research. We will omit discussion of PC arising from other gastrointestinal malignancies, low-grade mucinous appendiceal neoplasm (LAMN), and pseudomyxoma peritonei, given the markedly different clinical outcomes with these disease processes.

## 2. Background

### 2.1. The Peritoneum

In its simplest form, the peritoneum is a protective serous membrane covering the abdominal and pelvic organs that is composed of parietal and visceral layers. Both layers are embryologically derived from the lateral plate of the intraembryonic mesoderm. The parietal mesoderm develops into parietal pleura, pericardium, and parietal peritoneum, while the visceral mesoderm becomes visceral pleura, epicardium, and visceral peritoneum. Each layer is composed of a single sheet of mesothelial cells that sit on a basal membrane of connective tissue [12,13,14]. The area between each basal membrane forms the peritoneal space, which carries 5–100 mL of fluid that participates in homeostasis, tissue healing, and immunity [12]. The peritoneal space also serves as a conduit for vessels, nerves, and lymphatics. Folded areas of the peritoneum form ligaments, omenta, and mesenteries, including the falciform ligament and greater and lesser omenta. Diaphragmatic movements encourage peritoneal fluid circulation by creating hydrostatic pressure that draws fluid from the infra-mesocolic region to the supra-mesocolic region. Most fluid drains via lymphatic stomata along the diaphragm, with a concentrated focus on the right hemidiaphragm. A minority of fluid circulates into the abdominal cavity by stomata located on the greater and lesser omenta [12,13,14]. This physiological flow of fluid explains why cancers easily disseminate throughout the peritoneal cavity and readily form deposits along the diaphragm and omentum (i.e., “omental cake”) [13,14,15].

The parietal peritoneum receives its blood supply from the abdominal wall anteriorly and directly from the abdominal aorta posteriorly. The visceral peritoneum blood supply is from the celiac and superior and inferior mesenteric arteries and drains into the portal vein. This drainage pathway is important because any medications absorbed through the peritoneum, including those used during HIPEC, are subject to a first-pass hepatic metabolism. However, despite a rich vascular network, lymphatics constitute the majority of the peritoneal space. In fact, only 1–2% of cardiac output reaches the peritoneum [13]. This limits the delivery of systemic therapies to peritoneal tumors and explains why PC historically has a poor response to traditional intravenous (IV) chemotherapies [13,14,15,16].

### 2.2. Clinical Presentation of PC

Patients with PC often present with nonspecific symptoms, ranging from asymptomatic in early disease to surgical emergencies in advanced cases. Typical symptoms are nonspecific and include bloating, anorexia, back pain, abdominal pain, and urinary changes [2,17]. Patients may have occult progression to advanced disease because of the vague nature of symptoms and difficulty in the early diagnosis of PC [7]. It is common for patients to be diagnosed with PC at the time of surgery. With more advanced disease, patients may present with new-onset ascites, intestinal obstruction, and less commonly with perforation and fistula formation. Patients with histories of cancers known to metastasize to the peritoneum are more likely to present with advanced disease with symptoms as the first sign of recurrence.

### 2.3. CRS and HIPEC Beginnings

PC was first described in 1908 by Miller and Wynn [18]. Prior to the implementation of systemic chemotherapy, patients with PC from GI cancers survived <1–7 months [19]. With the introduction of 5-fluoruracil (5-FU) in the 1950s, survival increased to 6–12 months [2,17,20,21]. In the last two decades, these outcomes have improved to approximately 30 months with the introduction of oxaliplatin, irinotecan, and targeted therapies [22]. The concept of cytoreduction or the macroscopic removal of peritoneal tumors to reduce cancer burden was first established by Meigs for ovarian cancer in 1934 [23]. Later, in 1955, Weissberger pioneered the practice of directly treating peritoneal tumor deposits with intraperitoneal chemotherapy [24]. Despite recognition as a unique disease process with ineffective treatment options, surgical debulking and intraperitoneal chemotherapy for PC did not become a well-accepted treatment modality until the late 1970s to 1980s when interest, research, and trials for these modalities received increased attention. In 1978, Dedrick et al. established that the direct exposure of cytotoxic drugs to tumor tissues penetrated only 1–3 mm, demonstrating the necessity of removing macroscopic lesions for intraperitoneal chemotherapy to be effective [25]. During the same time period, Larkin et al. demonstrated that whole-body thermotherapy to 42 °C (108 °F) reduced tumor burden in advanced solid tumors [26]. Additional studies explored regional thermotherapy and various delivery methods for intraperitoneal chemotherapy [27,28,29]. Spratt et al. combined these advances in thermotherapy and intraperitoneal chemotherapy and performed the first iteration of combined CRS and HIPEC with a thermal transfusion infiltration system [28]. In 1988, Fujimoto et al. provided evidence that heat increased the efficacy of select chemotherapies [30], thereby providing a mechanistic rationale to combine heat with cytotoxic chemotherapy during HIPEC.

In 1995, Sugarbaker established a complete peritonectomy with six procedures for surgical debulking for PC. Complete peritonectomy included greater omentectomy with splenectomy, lesser omentectomy with cholecystectomy, omental bursa omentectomy, antrectomy, right and left upper quadrant omentectomies, and pelvic peritonectomy with sigmoid resection [31]. Subsequent studies in the 1990s–2000s demonstrated that complete cytoreduction promoted the maximal efficacy of intraperitoneal therapies; without complete cytoreduction, the median survival averaged 6 months, similar to the outcomes observed without surgical intervention [32,33,34,35,36,37,38,39,40]. More recent studies have shown that complete removal of macroscopic disease without completing the six peritonectomy procedures in the absence of disease was sufficient for survival benefit with CRS and have since redefined the term “complete cytoreduction” [41,42]. Specifically, in PC arising from appendiceal and CRC, recent studies have shown that with combined modern systemic therapeutic regimens and CRS/HIPEC survival averages 30 months and can reach over 60 months [36,43,44,45,46,47].

## 3. Prognostic Indicators

### 3.1. Preoperative Patient Selection

Given the physiological toll that CRS/HIPEC extracts and the potential for adverse outcomes, various risk-stratification tools have been developed to determine which patients with PC will derive benefit from this intervention. Those techniques demonstrating efficacy for appendiceal and CRCs are described.

#### 3.1.1. Prior Surgical Score (PSS)

The majority of patients with PC will have procedures prior to undergoing CRS/HIPEC in order to obtain a formal diagnosis of malignancy and/or PC. The PSS quantifies the level of prior surgical intervention to estimate the overall adhesive disease that is likely to be encountered during CRS/HIPEC. The PSS is calculated by separating the abdomen into nine regions. The PSS is as follows: 0, no prior surgery or only a biopsy has been performed; 1, exploratory laparotomy with dissection of only one region; 2, exploratory laparotomy with dissection of two to five regions; and 3, laparotomy with extensive cytoreduction in >five regions [48]. Prior extensive surgical treatment is associated with delayed CRS/HIPEC and worse progression-free and overall survival for patients with appendiceal mucinous adenocarcinoma. However, no benefit has been found when using PSS for CRC [48,49,50].

#### 3.1.2. Peritoneal Carcinomatosis Index (PCI)

In 1996, Sugarbaker and colleagues described the PCI to quantify peritoneal disease burden and determine patients who would benefit from CRS [51,52]. The PCI is the sum of scores from nine abdominopelvic regions and four small intestinal regions. Each region is assigned a score between 0–3 based on the largest tumor size, with a maximum score of 39. Regions are assigned scores as follows: 0, no tumor deposit; 1, largest tumor measures <0.5 cm; 2, largest tumor measures 0.5 to <5.0 cm; and 3, largest tumor ≥ 5.0 cm or confluence of disease. Although originally developed for intraoperative staging, with the drastic improvement and decreased costs of modern imaging techniques, PCI scores calculated with preoperative imaging have since been shown to be comparable to surgical PCI scores [7,53]. Although magnetic resonance (MR) may be more accurate than computed topography (CT) in calculating PCI scores, it is important to note that diagnostic laparoscopy is still commonly used for inconsistent or equivocal imaging [7,11,53,54,55]. The PCI score is now accepted as an independent risk factor for morbidity and mortality for patients undergoing CRS/HIPEC for appendiceal and colorectal malignancies [56,57]. In general, lower PCI scores confer more favorable outcomes. For example, worse outcomes have been observed with CRS/HIPEC in patients with PCI >19 for appendiceal and CRC [46,58,59].

#### 3.1.3. Peritoneal Surface Disease Severity Score (PSDSS)

Sugarbaker established the PSDSS in 1998 to stratify patients without the need for intraoperative staging [60]. Given the limitations of imaging techniques at the time, the PSDSS combines the PCI score obtained by preoperative imaging, clinical symptoms, and histology to preoperatively calculate the resectability of peritoneal disease with CRS. Modern studies have demonstrated the utility of using the PSDSS for mucinous appendiceal neoplasms, but the additional variables in the PSDSS vs. the PCI have not been shown to accurately predict outcomes for patients with metastatic CRC [61,62,63,64]. Given these limitations, the PCI score is more frequently used clinically.

### 3.2. Predicting Postoperative Outcomes

As with most cancers, complete tumor resection has been shown to improve outcomes for patients with PC. Two systems defining surgical margins are commonly used in clinical practice and throughout the literature. Neither has been found to be superior.

#### 3.2.1. Completeness of Cytoreduction (CC) Score

In 1994, Sugarbaker described the completeness of cytoreduction (CC) score to quantify residual disease after CRS [65]. No residual disease is scored as CC-0, tumors < 0.25 mm as CC-1, residual disease of 0.25 mm–2.5 cm as CC-2, and nodules > 2.5 cm as CC-3. Complete cytoreduction is associated with improved survival. Contrary to prior practice defining complete cytoreduction as CC-0 or CC-1, various recent studies have demonstrated survival benefits with CC-0 over CC-1 resections. Complete cytoreduction for metastatic appendiceal and CRCs is now attained only with CC-0 [32,66,67,68].

#### 3.2.2. Resection (R) Score

The American Joint Committee on Cancer staging manual provides an alternative cytoreduction completion scoring system to the CC score. This classification subdivides complete macroscopic removal into negative and positive microscopic disease with R0 and R1, respectively, based on pathological margins or cytology. For residual macroscopic disease, R2a is described as residual tumors < 0.5 cm, R2b designates tumors between 0.5–2.0 cm, and R2c represents remaining nodules > 2 cm. Similar to the CC score, R0 and R1 resections are associated with improved survival [33,36,69,70].

### 3.3. Emerging Predictive Modalities

Cell-free circulating tumor DNA (ctDNA) has been shown to be an effective biomarker to detect various cancers. Early studies have shown encouraging results using preoperative ctDNA levels to enhance surgical eligibility decision making and postoperative monitoring to detect disease recurrence that may be difficult to detect early on imaging [71,72]. However, similar to circulating tumor markers, circulating ctDNA levels do not consistently correlate with peritoneal tumor burden [71,72,73]. Comparably, immunoprofiling has been proposed for pre- and post-operative disease detection and potential therapeutic selection with promising results [74,75,76]. Prospective studies evaluating the prognostic implications of using ctDNA, new tumor markers, and immunoprofiling before and after intraperitoneal chemotherapy methods are underway (NCT04122885, NCT04083547).

## 4. HIPEC Techniques

### 4.1. Open HIPEC

Multiple techniques for HIPEC have been described since its inception over 40 years ago. The open or “colosseum” technique was first described by Sugarbaker in 1995 and involves elevating the skin edges with sutures or retractors, the placement of closed suction drains, and covering the open abdomen with a plastic sheet with an opening for the surgeon’s arm to manually agitate, shake, and circulate heated chemotherapy to reach all abdominopelvic surfaces [31,65].

### 4.2. Closed HIPEC

Due to concerns with the aerosolization of chemotherapy and the safety of intraoperative staff [77], the closed technique is now more commonly utilized and involves a temporary abdominal closure after securing in-flow and out-flow catheters. The abdomen is then manually agitated by external abdominal massage [31]. A recent retrospective study comparing the open and closed techniques by the US HIPEC Collaborative in 2020 found that the choice of open or closed HIPEC techniques did not correlate with patient outcomes [78]. Other emerging adjunctive techniques for use during CRS/HIPEC include the addition of agents to evaluate vessel and tumor presence to improve the rates of complete cytoreduction (NCT04950166, NCT03517852).

### 4.3. Laparoscopic HIPEC

In addition to diagnostic laparoscopy to determine the extent of peritoneal disease, laparoscopic CRS/HIPEC techniques are used with palliative and curative intent to reduce morbidity, facilitate faster recovery, and shorten hospital stays. In 2017, the American Society of Peritoneal Surface Malignancies (ASPSM) demonstrated that laparoscopic HIPEC techniques were safe and feasible in patients with PCI < 11 [79]. The Peritoneal Surface Oncology Group International (PSOGI) demonstrated similar safety and efficacy findings in 2020 [80]. Additional studies continue to show favorable surgical outcomes and long-term disease remission with minimally invasive HIPEC for patients with low tumor burdens [81,82,83,84,85,86,87,88,89,90,91].

## 5. HIPEC Safety

Once considered a highly risky procedure with mortality ranging from 0–17% and morbidity up to 60% [92], CRS/HIPEC has been shown to be safe, with a similar or better risk profile compared to other major cancer operations when appropriate patients are selected [1,44,93,94]. In fact, over the last two decades, CRS/HIPEC-related mortality now averages 0–4%, with most high-volume centers reporting 0–1% [1,44,93,94,95]. As with other large oncologic surgeries, the quality of life for patients immediately following CRS/HIPEC decreases. However, 3–6 months after surgery, patients show improved quality of life and survival benefits [95]. These improvements in HIPEC outcomes are multifactorial. Patient selection based on age, tumor burden, baseline functional status, the presence of ascites and weight loss, and recurrent disease have enabled the maximization of curative-intent CRS/HIPEC with limited morbidity and mortality [46,96,97,98,99,100,101]. Improved surgical techniques and protocols have also contributed. However, there is a steep learning curve for facilities and surgeons performing CRS/HIPEC. Increased system and surgeon experience and numbers performing CRS/HIPEC have further improved morbidity and mortality rates [102,103].

In 2016, the NCCN modified its recommendations for CRC with peritoneal disease from HIPEC only in the setting of clinical trials [104] to recommending HIPEC when performed at experienced centers [105]. The Chicago Consensus Working Group on peritoneal surface malignancies released similar updated guidelines in 2018, recommending HIPEC be performed at high-volume experienced centers [106]. Indeed, it has been shown that high-volume centers, averaging 55 cases per year, can have as much impact on patient outcomes as individual surgeon volume [107,108]. These effects are likely due to technical experience in performing multivisceral organ resections at both the system and surgeon levels. It is not surprising that a steep learning curve remains for centers and surgeons to perform CRS/HIPEC that result in favorable operative and oncologic outcomes [102,103]. Studies have shown that centers require approximately 100–180 cases to gain HIPEC proficiency at a system level [102,103,109]. However, surgeons require at least 90 and up to 220 cases to become CRS/HIPEC proficient, which can take 10 years for an individual surgeon to reach [102,107,108,110]. To this end, the Chicago Consensus Working Group established standards for facilities to become specialized in treating peritoneal surface diseases, including institutions maintaining at least two surgeons experienced with CRS/HIPEC, surgeons performing a minimum number of CRS/HIPEC procedures during training and per year while in practice [106].

Despite decreased mortality, morbidity following HIPEC can still reach 30% [44,94]. The most common postoperative complications include anastomotic leaks, bleeding, pulmonary complications, and deep and soft-tissue infections [1,44,93,94]. In fact, red blood cell (RBC) transfusion rates during and following HIPEC can range from 25–74% [1,44,93,111,112,113]. Additional studies have associated perioperative CRS/HIPEC RBC transfusions with worse oncologic, surgical, and survival outcomes in CRS/HIPEC [112,114,115,116]. However, preoperative RBC, clotting factor, and TXA transfusions have been shown to limit perioperative RBC transfusions and do not confer the same unfavorable outcomes as perioperative transfusions [117,118,119,120]. In a preliminary retrospective evaluation of coagulopathy in our CRS/HIPEC patients, we identified abnormal coagulation parameters preoperatively and pre-HIPEC, and significant changes in these values following HIPEC, consistent with prior studies [121,122]. To evaluate the impact these findings have on patient outcomes, we have initiated a standard protocol for monitoring coagulopathy before, during, and after HIPEC. The results from our prospective investigation will be timely, given similar studies evaluating the use of thromboelastography (TEG)-guided resuscitation during and after HIPEC (NCT03956836) [123]. Our goal is to identify whether patients with preoperative and perioperative coagulopathy and anemia require more RBC transfusions than other HIPEC patients. If so, a protocol to preoperatively correct anemia and coagulopathy with targeted transfusions in patients undergoing HIPEC may be of benefit since these interventions have not been shown to confer negative postoperative outcomes.

## 6. Landmark Efficacy Studies

### 6.1. HIPEC with Curative Intent

A landmark randomized controlled trial (RCT) evaluating CRS/HIPEC was published in 2003 [124]. Verwaal et al. compared standard-of-care systemic chemotherapy (n = 51) to CRS/HIPEC (n = 54) in patients with PC from appendiceal or CRC. Systemic therapy included 5-FU + leucovorin for 26 weeks or irinotecan for 6 months (if patients had received 5-FU treatment within the previous 12 months). Patients in the chemotherapy group underwent surgery for intestinal obstruction with diversion with bypass or stoma. CRS/HIPEC patients received mitomycin C (MMC) for 90 min and standard adjuvant systemic therapy. Complete cytoreduction was achieved in 41% of patients. Compared to control, patients who underwent CRS/HIPEC demonstrated an overall survival (OS) of 22.2 months vs. 12.6 months in the chemotherapy arm. Surgical mortality associated with CRS/HIPEC was 8%, which was consistent with contemporary studies. However, overall mortality was equal between the two cohorts for the first 6 months. Although this study was the first to definitively demonstrate a survival benefit favoring CRS/HIPEC, there were substantial limitations. Notably, patients with extensive PC were included in both cohorts. The study noted that these patients had poorer outcomes regardless of treatment, giving one of the first reports that disease burden could predict long-term outcomes from CRS/HIPEC. Furthermore, only a minority of patients in the CRS/HIPEC arm had complete cytoreduction. A subanalysis suggested that achieving complete cytoreduction was required for improved outcomes, supporting data that showed a complete macroscopic eradication of disease is associated with optimal outcomes. In 2008, long-term results from the study were released and continued to show that patients with complete cytoreduction maintained a survival benefit. In fact, the 5-year survival was 45% with complete cytoreduction but only 8–10% for incomplete cytoreduction or systemic therapy alone, demonstrating that surgical intervention resulting in incomplete cytoreduction offered no survival benefit over systemic therapies [125].

A more recent multicenter RCT released in 2021, PRODIGE 7, did not show a benefit of CRS/HIPEC using high-dose oxaliplatin for 30 min over CRS alone [126]. Although this study demonstrated a substantial improvement in overall survival, over 40 months in both groups, this study had many shortcomings that mandate a cautious interpretation of these study results. First, the study did not meet its primary endpoint and was not powered for hypothesis testing. Notably, the study included patients who received incomplete cytoreduction with CC-1 resections, which is an established factor that directly impacts survival. In the control group, 12% of patients crossed-over to receive subsequent CRS/HIPEC due to isolated peritoneal recurrence. However, no patients in the CRS/HIPEC group received secondary CRS/HIPEC. These differences are difficult to control with the intent-to-treat analysis used in this study.

Additionally, the majority of patients in the study were heavily pre-treated with oxaliplatin-based therapies, which may have resulted in peritoneal tumors resistant to oxaliplatin-based HIPEC, thus resulting in less effective disease control from HIPEC therapy [127]. Moreover, the carrier fluid (D5W), dosage of oxaliplatin (360–460 mg/m^2^), and lack of concomitant IV 5-FU during HIPEC call into question the efficacy of the HIPEC treatment in this study, which does not follow current US guidelines (Table 1). Furthermore, the adjuvant systemic therapy used in this study consisted only of 5-FU and leucovorin, which is inconsistent with current US standards-of-care [128]. A lack of adherence to current US treatment guidelines limits the applicability of these study results to the US patient population. Additionally, patients with high PCI scores (20–25) were included despite lower PCI scores previously demonstrating an enhanced benefit from HIPEC. In fact, subgroup analyses showed that patients with PCI scores between 11–15 had improved survival. However, because 25% of the patients included in the study had a PCI > 15, the overall survival findings remain unclear. Additional factors raise questions about the accuracy and validity of this study. Patients in the HIPEC intervention arm experienced more post-operative complications compared to the control arm: 42% vs. 32% at 30 days and 26% vs. 15% at 60 days, respectively. Furthermore, the mortality rates were 3% for the HIPEC group and 2% for the control group, both above the established average of <1% for high-volume CRS/HIPEC centers, such as those included here. Altogether, these limitations create an unclear picture requiring restraint when interpreting these study results.

### 6.2. HIPEC for Palliation

PC recurrence following CRS/HIPEC is common, even with CC-0 resections, particularly in patients with high PCI scores and unfavorable histologies. In fact, up to 46% of colorectal and 100% of perforated appendiceal cancers may develop recurrent peritoneal disease despite multimodal therapies [133,134,135]. In conditions where CRS/HIPEC is not expected to be curative, such as those with PCI > 19 and CRC with ascites, CRS/HIPEC can provide palliation for debilitating symptoms and improve quality of life. Multiple retrospective studies have shown that HIPEC can successfully control malignant ascites in up to 95% of patients with underlying GI malignancies [83,84,136,137,138,139,140]. Furthermore, because complete cytoreduction is not feasible in these patients, laparoscopic HIPEC is a reasonable approach with less associated morbidity [83,137,138,139,140,141,142]. Indeed, the Chicago Consensus Working Group now recommends laparoscopic HIPEC for the management of malignant ascites in patients who are not candidates for curative-intent CRS/HIPEC [143].

### 6.3. HIPEC for Prevention of Disease Recurrence

In patients at risk of developing PC, two proactive approaches have been proposed: (1) prophylactic HIPEC at the time of primary tumor resection and (2) second-look surgery including HIPEC. The COLOPEC trial published in 2019 investigated prophylactic HIPEC in patients undergoing oncologic resection of T4 or perforated CRC without PC [144]. The PROPHYLOCHIP (PRODIGE 15) trial evaluated the use of HIPEC during routine second-look surgery after complete CRS in patients with CRC [145]. However, there were significant limitations with both of these studies, and the lack of benefit is contested because these studies also suggest a potential benefit from HIPEC in the setting of macroscopic disease. However, like the PRODIGE 7 trial, both studies had design flaws limiting the applicability of their results.

The COLOPEC trial was confounded by the fact that 9% of patients in the prophylactic HIPEC arm were found to have PC shortly after surgery, indicating synchronous metastasis that were missed during surgery. These patients did not receive prophylactic HIPEC or complete CRS prior to HIPEC, which is a major contributing factor to survival, as discussed previously. Furthermore, patients in this study had HIPEC at the time of traditional oncologic resection (up to 10 days after surgery) or 5–8 weeks after surgery, thereby introducing confounding effects based on the timing of HIPEC. Additionally, similar to the PRODIGE 7 trial, the utilized HIPEC regimen was 460 mg/m^2^ oxaliplatin for 30 min, which differs from the standard US HIPEC recommendations (Table 1). Although IV 5-FU and leucovorin were administered concomitantly with intraperitoneal oxaliplatin, similar to US protocols with oxaliplatin-based HIPEC, this regimen is not the first-line standard-of-care HIPEC procedure in the US and it results in data that are not directly applicable to the US population.

In the PROPHYLOCHIP trial, patients with PC from CRC underwent traditional oncologic resection plus CRS. However, contrary to common standard practice, HIPEC was not performed in these patients following CRS during the initial surgery. In the investigative arm, second-look surgery was performed after the completion of 6 months of standard systemic chemotherapy. HIPEC regimens included: 460 mg/m^2^ oxaliplatin for 30 min with IV 400 mg/m^2^ 5-FU, 300 mg/m^2^ oxaliplatin + 200 mg/m^2^ irinotecan for 30 min with 400 mg/m^2^ 5-FU IV, or 35 mg/m^2^ MMC for 30 min for patients with previous neurotoxicity from oxaliplatin. The control arm included adjuvant systemic therapy alone. Again, as with the PRODIGE 7 trial, the use of non-standard and oxaliplatin-based HIPEC regimens in the majority of patients calls into question the efficacy of HIPEC performed in this study. Although peritoneal disease recurrence was lower in the investigational arm of this study, perhaps due to unintentional under-staging by utilizing preoperative imaging alone and, therefore, a higher portion of patients with an intermediate risk of PC recurrence, the 3-year disease-free survival was not significantly different.

### 6.4. Alternative Intraperitoneal Chemotherapy Techniques 

**Figure 1:**
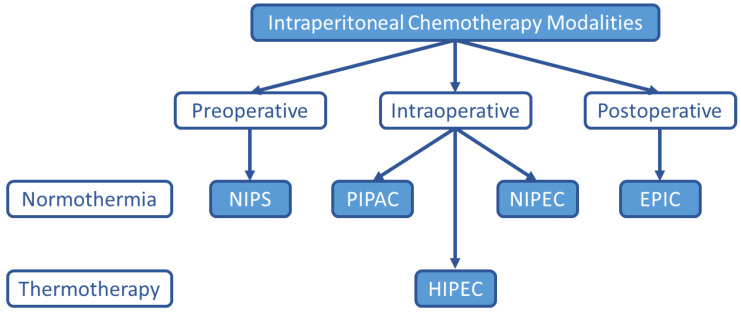
**Alternate Intraperitoneal Chemotherapy Treatment Modalities.** Although HIPEC is the current standard-of-care intraperitoneal chemotherapy treatment option for PC from appendiceal and colorectal malignancies, other intraperitoneal chemotherapy treatment strategies are under investigation. NIPS (neoadjuvant intraperitoneal and systemic chemotherapy), PIPAC (pressurized intraperitoneal aerosol chemotherapy), HIPEC (hyperthermic intraperitoneal chemotherapy), NIPEC (normothermic intraperitoneal chemotherapy), EPIC (early postoperative intraperitoneal chemotherapy).

Although HIPEC remains the most common intraperitoneal chemotherapy treatment option for PC, many alternatives have been explored and are under investigation as listed in Figure 1.

#### 6.4.1. Early Postoperative Intraperitoneal Chemotherapy (EPIC)

In 1990, Sugarbaker initially described EPIC, which included intraperitoneal chemotherapy administered on postoperative days 1 and 5 following CRS [146]. In an RCT published in 2016, Cashin and colleagues found that the effect of CRS/EPIC was superior to systemic chemotherapy alone for CRC with PC [147]. However, more recent studies evaluating the utility of adding EPIC to CRS/HIPEC regimens show increased morbidity and surgical complications compared to CRS/HIPEC without EPIC, and survival benefits are unclear between the two regimens [148,149,150,151]. Concerns of increased perioperative morbidity, the development of increased postoperative adhesions, an incomplete distribution of chemotherapy throughout the abdomen and pelvis in the perioperative setting, and unanswered questions surrounding EPIC efficacy compared to HIPEC have resulted in EPIC falling out of favor [146,152,153,154]. The ICARuS study is an important ongoing phase 2 RCT that will directly compare CRS/HIPEC vs. CRS/EPIC and will hopefully answer questions regarding which method offers superior efficacy (NCT01815359). Additionally, for difficult-to-treat cancers with poor responses to current systemic therapy and CRS/HIPEC regimens, long-term EPIC regimens have demonstrated longer-term benefits with additional studies underway (NCT05056389) [155,156].

#### 6.4.2. Normothermic Intraperitoneal Chemotherapy (NIPEC)

Given continued high PC recurrence rates following current CRS/HIPEC regimens, NIPEC has been proposed in an effort to enable the utilization of non-thermostable chemotherapies that may result in improved efficacy and outcomes [130,157]. However, few trials have directly investigated the efficacy of intraoperative NIPEC vs. standard HIPEC regimens. A 2001 RCT by Yonemura et al. demonstrated that the synergistic cytotoxicity observed with combined hyperthermia and MMC provided a significantly better 5-year survival compared to NIPEC for patients with gastric cancer (61% vs. 44%) [158]. However, a meta-analysis in 2012 showed no benefit of NIPEC over HIPEC for gastric cancer [159]. A study comparing HIPEC to NIPEC is underway for ovarian cancer [160]. Historical and future prospective studies evaluating NIPEC for other cancer types, including appendiceal and CRC, are lacking.

#### 6.4.3. Pressurized Intraperitoneal Aerosol Chemotherapy (PIPAC)

First proposed in 2011, PIPAC takes advantage of the increased intra-abdominal pressure created during laparoscopy to deliver aerosolized particles to residual tumor cells, which promotes tissue penetration and, thereby, the efficacy of intraperitoneal chemotherapy [161,162]. This principle has been theorized to overcome the chemoresistance observed with some HIPEC agents [163]. Further studies have established favorable pharmacokinetic and safety profiles, efficacy for isolated refractory PC, and ease of repeated administration [161,162,164,165,166,167,168]. However, few studies have directly compared HIPEC to PIPAC. One recent pharmacokinetic study demonstrated that PIPAC and HIPEC resulted in similar systemic and parietal peritoneal absorption, but visceral peritoneal absorption was significantly higher in the PIPAC group, suggesting improved penetration of chemotherapy into tissues [169]. Pre-clinical studies have suggested a combined HIPEC + PIPAC technique may take advantage of synergistic cytotoxicity and improved tumor penetration with hyperthermia during PIPAC [170]. Further studies are required to evaluate the efficacy of HIPEC compared to PIPAC.

#### 6.4.4. Neoadjuvant Intraperitoneal and Systemic Chemotherapy (NIPS)

Combined with systemic neoadjuvant therapies, neoadjuvant intraperitoneal chemotherapy aims to reduce the overall peritoneal tumor burden to make CRS feasible and improve rates of complete cytoreduction [167]. For ovarian, gastric, and CRCs, NIPS has shown low morbidity and mortality and indeed shown higher rates of complete cytoreduction [167,171,172]. Yonemura and colleges described the survival benefit of using NIPS for gastric cancer [173,174]. Conversely, the PHEONIX trial failed to demonstrate the superiority of NIPS vs. systemic therapy alone for gastric cancer [175]. However, this trial was underpowered for statistical results and enrolled more patients with ascites, a known poor prognostic factor, in the NIPS intervention group. Additionally, a subanalysis revealed that neoadjuvant intraperitoneal chemotherapy without systemic IV chemotherapy may offer survival benefits. Since then, retrospective studies have suggested the superiority of NIPS over systemic therapy alone [171,176,177]. Additional studies evaluating NIPS with non-traditional systemic chemotherapies are underway [177]. Survival outcomes following NIPS remain mixed for cancers other than gastric, however, and require further prospective studies [167,178]. One upcoming trial aims to evaluate the effect of neoadjuvant systemic chemotherapy and PIPAC prior to CRS/HIPEC for CRC (NCT04475159). The Dragon II trial aims to evaluate NIPS using laparoscopic HIPEC prior to curative-intent CRS/HIPEC for gastric cancer [179]. These studies will help to determine if neoadjuvant intraperitoneal chemotherapy should be incorporated into future recommendations.

### 6.5. HIPEC Agents and Adjunctive Therapies

#### 6.5.1. Current Regimens

Ideal HIPEC agents have a large molecular weight, demonstrate stability and synergistic effects with heat, have a large peritoneal to plasma area-under-the-curve (AUC) ratio demonstrating limited systemic toxicity, and show antitumor efficacy against the tumor of interest [130]. Commonly used HIPEC agents meeting these criteria include MMC, cisplatin, oxaliplatin, paclitaxel, doxorubicin, and 5-FU [128,130]. However, the numerous aforementioned requirements have limited the development and evaluation of new HIPEC therapies, resulting in little evolution in HIPEC regimens for decades. MMC, the most commonly used agent, was discovered in the 1950s and has been in use with HIPEC since the 1980s [180]. In 2002, Elias et al. described bidirectional HIPEC with concomitant IV 5-FU to potentiate the cytotoxicity of oxaliplatin, the second most common HIPEC agent, by targeting tumors from the visceral and parietal sides [181]. These results were supported by additional groups and changed the standard recommendations for oxaliplatin-based HIPEC [182].

Since that time, however, there have been little to no changes to HIPEC regimens, resulting in different recommendations worldwide. For instance, for appendiceal and CRC-derived PC, MMC is preferred over oxaliplatin-containing regimens in the US, while oxaliplatin regimens are favored in Europe, which is reflected in how historical and emerging HIPEC studies are developed and carried out in each region [183]. The multitude of alternative HIPEC regimens has resulted in clinical studies with fundamentally different designs that limit the applicability of results to patients in other regions [183]. Of the limited data available, one RCT in mucinous appendiceal cancer demonstrated that patients with baseline thrombocytopenia may benefit from MMC, while those with baseline leukopenia may benefit from oxaliplatin [184]. Neither agent was found to have superior outcomes, however.

Additional retrospective analyses have shown mixed results when evaluating the efficacy and safety profiles between these commonly used agents when treating CRC, but more studies demonstrate equivalent efficacy [185,186,187,188,189,190,191,192,193,194,195,196]. Further studies comparing oxaliplatin vs. MMC + doxorubicin [197], irinotecan vs. oxaliplatin [198], oxaliplatin ± irinotecan [181,199], and MMC vs. melphalan [200] have been performed but have not yielded a definitively superior regimen. Upcoming trials directly comparing commonly used HIPEC regimens include: oxaliplatin ± irinotecan (NCT04861558), oxaliplatin vs. oxaliplatin + 5FU + irinotecan (NCT04861558), oxaliplatin vs. MMC vs. cisplatin + doxorubicin vs. cisplatin + MMC (NCT04847063). Despite these necessary forthcoming studies, comparisons of HIPEC agent doses are also lacking. Intra-regional regimen preferences between weight-based and flat dose regimens are common (Table 1). To this end, our institution is preparing to study the safety and efficacy of different commonly used MMC dosages used during HIPEC for appendiceal and CRC (NCT04779554). Altogether, the results from these essential trials will help to establish the most efficacious regimen(s) and aid in the development and adoption of standard international HIPEC recommendations.

#### 6.5.2. Emerging Therapeutics

Given the high frequency of PC recurrence, novel agents are required to make HIPEC more effective and establish curative treatment for those cancers that continue to harbor poor outcomes. New cytotoxic HIPEC agents under evaluation include MOC31PE [201,202], miRNA-409-3p [203], nanoparticle pegylated liposomal doxorubicin [204], Radspherin^®^ (NCT03732781), raltitrexed (NCT04761185), and lobaplatin (NCT04845490 and NCT04808466) [205,206]. Additionally, our group recently completed a phase 1 study evaluating the safety of nano-liposomal irinotecan (nal-Iri) during HIPEC for appendiceal and CRC (NCT04088786). Nal-Iri fulfills all requirements for optimal HIPEC agents, including thermostability, large molecular weight, low systemic absorption, and activity against CRC. CRC systemic therapy regimens including nal-Iri have demonstrated improved efficacy against chemo-resistant tumors, including traditional irinotecan [207]. The results from our dose escalation study (70–280 mg/m^2^) will lay the groundwork for upcoming phase 2 studies to evaluate nal-Iri efficacy in HIPEC for patients with appendiceal and CRCs.

The modern rise of novel immuno-oncologic therapies (IO) has impacted the treatment of many cancers, and PC is on the beginning path of these discoveries. CRS has itself been proposed to be a form of immunotherapy [208]. Subsequent studies have confirmed that tumor resection can re-activate the native immune system to an anti-tumor phenotype [209,210,211]. In fact, catumaxomab, a trifunctional antibody, was approved in Europe for intraperitoneal use to treat malignant ascites, has demonstrated promise to directly treat GI-derived PC, and is undergoing additional evaluation of efficacy for curative intent (NCT04222114, NCT01815528, and NCT01246440) [212,213,214,215,216]. Consequently, interest in intraperitoneal IOs has grown rapidly in recent years [217,218,219]. Upcoming and ongoing trials evaluating IOs as alternative intraperitoneal agents include: immune checkpoint inhibitors (ICIs), such as nivolumab ± ipilimumab (NCT03959761, NCT03508570, and NCT03172416), pembrolizumab (NCT03734692), and IMP321 (NCT03252938); TLR-3 agonists (NCT03734692); CAR-T and cytotoxic T-cell infusions (NCT03682744, NCT03323944, NCT03563326, NCT03907527, NCT02498912, and NCT03735589), IL-2 ± NK cell infusions (NCT04630769, NCT02976142, NCT02118285, and NCT03213964); immune modulators (NCT02219893); oncolytic viruses (NCT02759588 and NCT03663712); and personalized neoantigen vaccines (NCT03715985). Trials evaluating adjunctive IOs are also numerous and include the ICI camrelizumab (NCT04889768).

## 7. Summary of Recommendations and Conclusions

CRS/HIPEC has demonstrated substantial survival benefits for select patients with PC from appendiceal and CRCs (PCI < 20). These procedures should be performed at high-volume, multidisciplinary expert centers for optimal outcomes. Although CRS has undergone near universal standardization, HIPEC regimens remain diverse. Even recent consensus guidelines include various intraperitoneal chemotherapy regimens based on flat or weight-based dosing. Prospective studies supporting these regimens remain limited, and recent RCTs have produced contentious results. Carefully designed RCTs comparing current standard-of-care HIPEC regimens are needed to definitively identify superior regimens and are underway. Additional studies evaluating novel HIPEC agents, in particular IOs, are also desperately needed. Despite these limitations, for patients with few treatment options, such as those with PC from appendiceal and CRCs, CRS/HIPEC remains a valuable therapy that can prolong survival and quality of life.

## Figures and Tables

**Table 1:** **Recommended HIPEC Regimens for Appendiceal and Colorectal Cancers in the US.** The most common established regimens across societies are shown and first-line recommendations are bolded. MMC (mitomycin C), PCI (peritoneal carcinomatosis index).

Primary Malignancy	Agent	Duration	Dose/ Concentration	Bidirectional IV Therapy	Ref.
Appendiceal	**MMC**	**90 min**	**30 mg (0 min) + 10 mg (60 min)**	--	[129,130]
		90–120 min	30 mg/m^2^	--	[129,130]
	MMC + doxorubicin	90 min	15 mg/m^2^, 15 mg/m^2^	5-FU (400 mg/m^2^) + Leucovorin (20 mg/m^2^)	[129,130]
	Oxaliplatin	30 min	300 mg/m^2^	5-FU (400 mg/m^2^) + Leucovorin (20 mg/m^2^)	[129]
Colorectal	**MMC**	**90 min**	**30 mg (0 min) + 10 mg (60 min)**	--	[130,131,132]
		90–110 min	30 mg/m^2^	--	[130,131,132]
	Oxaliplatin (only with PCI 11–15)	30 min	300 mg/m^2^	5-FU (400 mg/m^2^) + Leucovorin (20 mg/m^2^)	[131]

## Data Availability

Not applicable.
